# Assessing the reliability and quality of content on social media regarding biologic therapies for psoriasis: A cross-sectional content analysis

**DOI:** 10.1371/journal.pone.0345570

**Published:** 2026-04-02

**Authors:** Jianghui Li, Jiaying Fu, Qin Sun, Huanhuan Lei, Jiayun Li, Qinye Li, Yu Wang, Jiashui Hu, Xiaoning Yan

**Affiliations:** 1 The First Clinical Medical College, Shaanxi University of Chinese Medicine, Xianyang, China; 2 Department of Dermatology, Shaanxi Hospital of Traditional Chinese Medicine, Xi’an, China; National Institutes of Health, University of the Philippines Manila / De La Salle University, PHILIPPINES

## Abstract

**Background:**

Biologic agents are an important novel therapeutic option for moderate-to-severe psoriasis. In recent years, TikTok and Bilibili have gradually become important channels for Chinese patients to obtain health information. This study aims to evaluate the content, quality, reliability, and transparency of videos related to biologic therapy for psoriasis on these platforms.

**Methods:**

We searched both platforms using the dual keywords “psoriasis” and “biological agents,” confirmed compliance with relevant criteria, and collected the top 150 videos based on their composite rankings. Fundamental characteristics, uploader categories, and content types were documented. Two independent reviewers evaluated video quality using the mDISCERN, GQS, and the JAMA criteria. Nonparametric tests were performed for group comparisons, and Spearman correlation analysis was applied.

**Results:**

Bilibili videos more frequently addressed medical expenses, types of biologic agents, and recurrence, whereas TikTok videos focused on etiology and clinical manifestations. The video quality was barely acceptable. On TikTok, the median GQS, mDISCERN, and JAMA scores were 3.00 (2.25, 4.00), 3.00 (3.00, 4.00), and 2.00 (2.00, 3.00). On Bilibili, the median scores were 3.00 (2.00, 4.00), 3.00 (2.00, 4.00), and 1.00 (1.00, 3.00). Videos uploaded by professional organizations achieved the highest GQS (median 4.00, IQR: 4.00–4.00) but had the lowest engagement. Engagement metrics showed a moderate correlation with quality scores (*P* < 0.05).

**Conclusions:**

This study found that videos related to biologic therapy for psoriasis lack content completeness, with overall quality, reliability, and transparency remaining at a suboptimal level. Greater participation by professional organizations and increased visibility of their videos should be encouraged to promote the dissemination of high-quality content. This study provides preliminary insights for health communication strategies and highlights the necessity of strengthening content regulation.

## Introduction

Psoriasis is a chronic, immune-mediated skin disease that is prevalent worldwide, with an estimated global prevalence of 2%–3% [1]. In China, the total number of psoriasis cases increased by 115.54% from 1990 to 2021, with a prevalence rate of approximately 474 per 100,000 people [2]. The quality of life of patients is substantially impacted by the long-term course and recurrent flare-ups of this condition [3,4]. Biologics have shown considerable benefits in enhancing therapy efficacy and patient adherence [5,6]. Nonetheless, elevated expenses, possible adverse effects, and obstacles in managing recurrence persist in complicating patients’ selection of treatment alternatives [7,8]. Therefore, increasing public awareness of biologic therapies for psoriasis is essential. Health education and information dissemination facilitate patient comprehension of treatment options, enhance adherence, and improve long-term outcomes, while also minimising misunderstandings and delays stemming from inadequate knowledge. This fosters standardised and optimised disease management universally [9,10].

In recent years, the continuous evolution of digital communication has enabled social media to gradually emerge as a major channel for patients to exchange treatment experiences and access medical information [11–13]. TikTok and Bilibili, the two most prominent Chinese short-video platforms, have significantly enhanced the rapid dissemination and extensive reach of health-related information due to their large user bases, intuitive content creation tools, and high levels of interactivity [14]. According to statistics, during peak periods, over 70% of people are active on at least one social media platform [15]. Nevertheless, concerns have been raised regarding the coverage, quality, and reliability of such content due to platform characteristics and audience diversity. Inaccurate information about diseases, including their causes, treatments, and biologics’ side effects, can cause the public to have false views about health, increase patient anxiety, erode patient-doctor confidence, and disturb the order of scientific communication [16]. Previous studies have demonstrated that short videos on topics such as atopic dermatitis, black skin and eczema, while achieving high levels of user engagement, generally exhibit suboptimal quality and reliability. Furthermore, no study has yet systematically evaluated short videos related to Psoriasis Biologics [17–19].

Consequently, this investigation implements a cross-sectional analysis to evaluate the quality and characteristics of pertinent videos on the TikTok and Bilibili platforms. The objective is to identify the strengths and weaknesses in the dissemination of medical information and to serve as a reference for clinical practice and patient education.

## Methods

### Data search and collection

**Fig 1:**
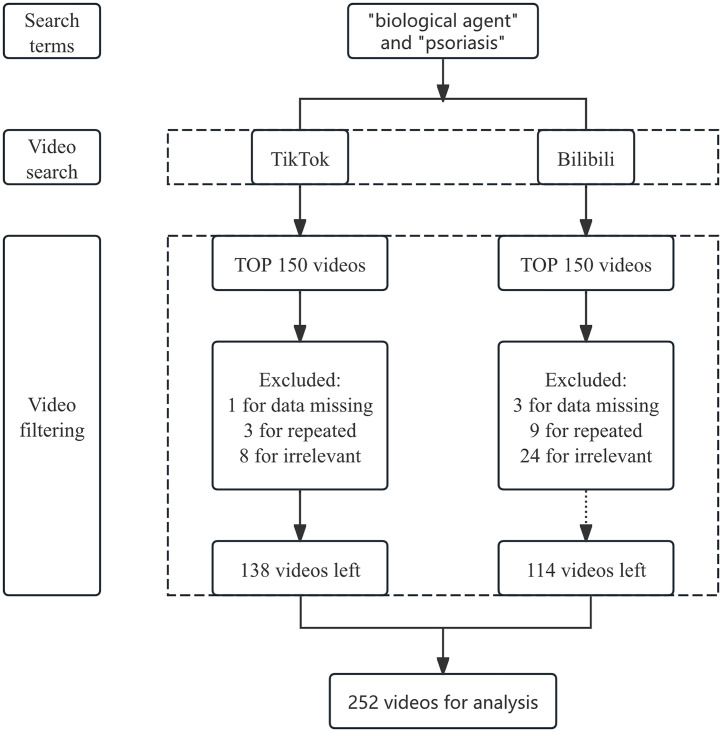
Video Collection Flowchart.

Simultaneous keyword searches for “biological agents” and “psoriasis” were conducted on TikTok and Bilibili platforms. Using default sorting rules, 150 videos were filtered and collected between August 27 and 28. Previous studies have shown that the top 100 videos are sufficient to represent all relevant videos in this field on each platform. To enhance accuracy, we expanded the sample size by 50 on each platform [20,21]. Data collection was conducted without the necessity of registering into accounts in order to reduce the potential bias from search history and personalised recommendations. To exclude: (a)videos with deleted accounts or lost content; (b) duplicate videos; (c) videos unrelated to biological agent treatment for psoriasis, a rigors screening process was implemented (Fig 1). After screening, a total of 252 relevant videos were ultimately evaluated. Researchers recorded and analysed the following essential parameters based on this framework: likes, comments, collections, shares, video content, and source classification. Epidemiology, etiology, clinical manifestations, diagnosis, types of biological agents, medical expenses, recurrence, and prevention were the eight categories into which video content falls. Source classification included three distinct categories: healthcare personnel, professional organizations, and individual users. All social media content in the study was publicly available at the time of data collection. It was sourced from the TikTok and Bilibili platforms, and the data collection and analysis methods fully complied with the terms and conditions of both platforms. All data are incorporated into the article and the supporting information (S1 Table).

### Video quality assessment

After standardised training, two independent researchers (J. FU & Q. SUN) assessed blinded utilising Global Quality Scale (GQS), Journal of the American Medical Association (JAMA) criteria and the modified DISCERN (mDISCERN) instrument, as in Tables 1–3 [22]. Double-blind evaluations prevented participants from seeing each other's ratings, ensuring study rigour. To resolve scoring conflicts (≥1-point difference in any assessment technique), a dermatologist (X. YAN) with over 30 years of experience was consulted to determine the final score.

**Table 1:** The Global Quality Score (GQS) quality criteria.

Item features	Points
Poor quality; poor flow of the videos; most information missing; not at all useful for patients	1
Generally poor quality; some information listed, but many important topics missing; of very limited use to patients	2
Moderate quality; suboptimal flow; some important adequately discussed, but other information poorly discussed; somewhat useful for patients	3
Good quality and generally good flow; most of the relevant information listed, but some topics not covered; useful for patients	4
Excellent quality and flow; very useful for patients	5

**Table 2:** The Modified DISCERN (mDISCERN) quality criteria.

Reliability Score
1. Is the video clear, concise, and understandable?
2. Are valid sources cited?
3. Is the content presented balanced and unbiased?
4. Are additional sources of content listed for patient reference?
5. Are areas of uncertainty mentioned?

* (1 point for answer ‘yes’, 0 point for answer ‘no’).

**Table 3:** The Journal of the American Medical Association (JAMA) benchmark criteria.

Score*	Score component	
1 score	Authorship	Author and contributor credentials and their affiliations should be provided.
1 score	Attribution	Clearly lists all copyright information and states references and sources for content.
1 score	Currency	Initial date of posted content and subsequent updates to content should be provided.
1 score	Disclosure	Conflicts of interest, funding, sponsorship, advertising, support, and video ownership should be fully disclosed.

*The criteria of each aspect were scored separately, and 1 point for each criterion with a total score of 4 points.

### Data analysis

The Shapiro–Wilk test was applied to assess the normality of continuous variables. Continuous variables not conforming to a normal distribution were presented as medians with interquartile ranges (IQR), whereas normally distributed continuous variables were expressed as mean ± standard deviation. Categorical variables were summarized as frequencies and percentages. For non-normally distributed continuous variables, the Mann–Whitney U test was employed for two-group comparisons, and the Wilcoxon rank-sum test was used for comparisons among three or more groups. For normally distributed continuous variables, comparisons between two groups were conducted using the independent samples t-test. Correlations between variables were examined using Spearman’s rank correlation analysis. GQS, mDISCERN, and JAMA scores were classified as ordinal variables, and inter-rater agreement was evaluated using the weighted Cohen's kappa coefficient, with κ > 0.80 deemed excellent. All tests were two-sided, and a *P*-value < 0.05 was considered statistically significant. All statistical analyses and graphical visualizations were performed using R software, version 4.4.2.

## Results

### Key features of videos on biologic therapy for psoriasis

**Fig 2:**
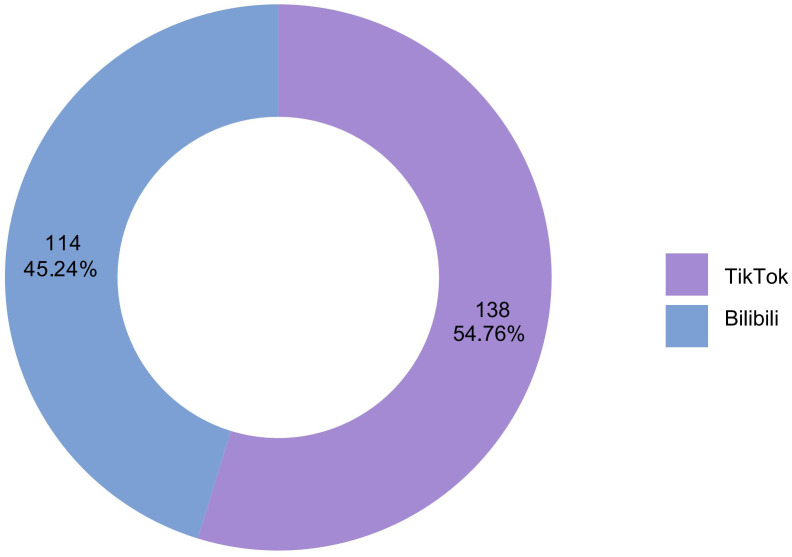
Proportion of Video Platforms Included.

We examined 252 videos from Bilibili and TikTok, with particular characteristics included in Table 4 and Fig 2. All TikTok group videos had superior interaction metrics, with the exception of video length. The Bilibili group consisted of 114 videos (45.24%), whereas the TikTok group included 138 videos (54.76%). Videos in the Bilibili group exhibited longer durations (96.50 seconds compared to 78.00 seconds), although the median number of likes in the Bilibili group was inferior to that of the TikTok group (6.00 versus 215.50), highlighting a considerable disparity between the two groups. In terms of bookmarks and comments, the Bilibili group exhibited lower medians compared to the TikTok group: 4.00 versus 49.00 and 1.00 versus 42.00, respectively. The identical trend persisted for shares (3.00 against 47.50). Table 5 summarizes the basic characteristics of the videos.

**Table 4:** Basic data of Biological Agents for Psoriasis Videos on TikTok and Bilibili.

Variables	TikTok	Bilibili	Total
**Basic Data**			
Videos	138	114	252
Video length(s)	15,897	29,967	45,864
Likes	83,793	42,052	125,845
Collections	31,850	16,977	48,827
Comments	24,314	9,148	33,462
Shares	32,390	7472	39,862

**Table 5:** Basic Characteristics of Biological Agents for Psoriasis Videos on TikTok and Bilibili.

Variables	TikTok (n = 138)	Bilibili (n = 114)	*P*
**General information**			
Video length(s), M (Q1, Q3)	78.00 (47.00, 123.75)	96.50 (56.25, 265.00)	**<0.01**
Likes, M (Q1, Q3)	215.50 (83.00, 529.25)	6.00 (2.00, 39.00)	**<0.001**
Collections, M (Q1, Q3)	49.00 (18.00, 183.00)	4.00 (1.00, 24.75)	**<0.001**
Comments, M (Q1, Q3)	42.00 (11.00, 130.75)	1.00 (0.00, 18.75)	**<0.001**
Shares, M (Q1, Q3)	47.50 (11.00, 149.25)	3.00 (0.00, 20.75)	**<0.001**

**Fig 3:**
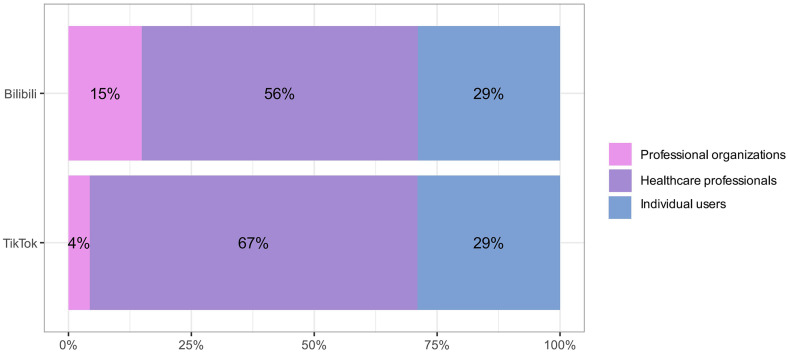
Proportion of Uploaders on Video Platforms.

Both groups mostly consist of healthcare professionals, with a greater proportion in the TikTok group (67%) than in the Bilibili group (56%). The percentage of individual users is the same at 29% for both groups. Professional organizations constitute 4% of the TikTok cohort and 15% of the Bilibili cohort (Fig 3).

**Fig 4:**
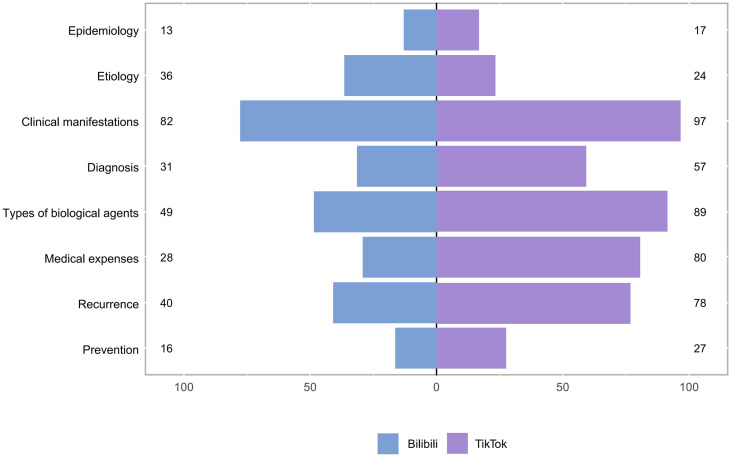
Comparison of Content Included in Platform Videos.

This study also evaluated the content covered in the videos, with Fig 4 and Table 6 illustrating this data visually. The TikTok group predominantly discussed clinical manifestations of psoriasis (70.29%), types of biological agents (64.49%), medical expenses (57.97%), recurrence (56.52%), and diagnosis (41.43%). Bilibili videos predominantly addressed clinical manifestations of psoriasis (71.93%), types of biological agents (42.98%), etiology (36.58%), recurrence (35.09%), and diagnosis (27.19%).

**Table 6:** Specific content of Biological Agents for Psoriasis Videos on TikTok and Bilibili.

Variables	TikTok (n = 138)	Bilibili (n = 114)
**Video content (n) (%)**		
Epidemiology	17 (12.32)	13 (11.40)
Etiology	24 (17.39)	36 (36.58)
Clinical manifestations	97 (70.29)	82 (71.93)
Diagnosis	57 (41.30)	31 (27.19)
Types of biological agents	89 (64.49)	49 (42.98)
Medical expenses	80 (57.97)	28 (24.56)
Recurrence	78 (56.52)	40 (35.09)
Prevention	27 (19.57)	16 (14.04)

### Comparison of mDISCERN, GQS, and JAMA scores across TikTok and Bilibili

The weighted Cohen's Kappa coefficients were GQS 0.83 (95% CI, 0.78–0.88), mDISCERN 0.89 (95% CI, 0.85–0.93), and JAMA 0.82 (0.76–0.87), indicating excellent agreement between the two reviewers. The disparities between the two platforms were predominantly apparent in mDISCERN and JAMA scores (*P* < 0.01). The mDISCERN score of TikTok videos was 3.00 (3.00, 4.00), whereas Bilibili videos scored 3.00 (2.00, 4.00). Regarding JAMA scores, TikTok achieved 2.00 (2.00, 3.00), while Bilibili scored 1.00 (1.00, 3.00). Other parameters are presented in Table 7.

**Table 7:** Quality, and Reliability of Biological Agents for Psoriasis Videos by Different Platforms on TikTok and Bilibili.

Variables	TikTok	Bilibili	*P*
mDiscern, M (Q1,Q3)	3.00 (3.00, 4.00)	3.00 (2.00, 4.00)	**< 0.01**
GQS, M (Q1,Q3)	3.00 (2.25, 4.00)	3.00 (2.00, 4.00)	0.084
JAMA, M (Q1,Q3)	2.00 (2.00, 3.00)	1.00 (1.00, 3.00)	**<0.001**

### Comparison of interaction metrics and quality scores among different uploaders

To enhance the analysis of video interaction data and quality scores across both platforms, we classified all video uploaders into three categories: professional organizations, healthcare professionals, and individual users (Table 8). An examination of video interaction metrics indicated notable disparities chiefly in video length and commentary among these groups. Videos generated by professional organizations exhibited the greatest duration, with a median of 247.00 seconds, succeeded by individual users at 149.00 seconds, and healthcare professionals at 59.00 seconds. Conversely, professional organizations’ videos received the fewest comments, with a median of only 2.00, while individual users garnered the most (73.00), followed by healthcare professionals (10.00).

**Table 8:** Characteristics, Quality, and Reliability of Biological Agents for Psoriasis Videos by Different Uploaders on TikTok and Bilibili.

Variables	Professional organizations (n = 23)	Healthcare professionals (n = 153)	Individual users (n = 76)	*P*
Video length(s), M (Q1,Q3)	247.00 (120.00,325.50)	59.00 (44.00,94.00)	149.00 (81.25,287.25)	**<.001**
Likes, M (Q1,Q3)	8.00 (2.50,234.50)	72.00 (6.00,333.00)	137.50 (14.75,437.25)	0.168
Collections, M (Q1,Q3)	4.00 (2.00,123.50)	22.00 (3.00,86.00)	26.00 (6.75,148.50)	0.316
Comments, M (Q1,Q3)	2.00 (0.00,32.50)	10.00 (1.00,46.00)	73.00 (9.75,299.25)	**<.001**
Shares, M (Q1,Q3)	4.00 (1.50,78.00)	12.00 (2.00,78.00)	26.00 (4.00,105.75)	0.689
GQS score, M (Q1,Q3)	4.00 (4.00,4.00)	3.00 (3.00,4.00)	3.00 (2.00,4.00)	**<.001**
mDISCERN score, M (Q1,Q3)	4.00 (3.00,4.50)	3.00 (2.00,4.00)	3.00 (2.00,4.00)	0.174
JAMA score, M (Q1, Q3)	3.00 (3.00,3.00)	2.00 (2.00,3.00)	2.00 (1.00,2.25)	**<.001**

**Fig 5:**
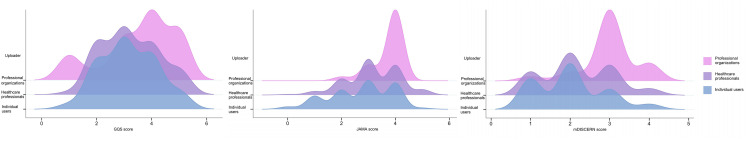
Scoring Trends Across Three Evaluation Criteria for Different Uploaders..

**Fig 6:**
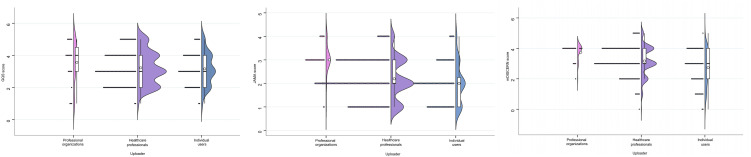
Score Distribution Across Three Rating Criteria for Different Uploaders. ns: not significant; *: *P* < 0.05; **: *P* < 0.005; ****: *P* < 0.00005.

In the video quality scoring analysis, differences among uploaders were primarily reflected in GQS and JAMA scores. The findings indicate that professional organizations achieved the highest GQS scores, while individual users received the lowest (4.00 vs. 3.00), demonstrating significant disparities in video quality. The JAMA scores reflected a similar trend: videos created by professional organisations showed greater standardisation, while individual users obtained the lowest scores (3.00 versus 2.00). The score distribution is illustrated in Fig 5, while the score differences are presented in Fig 6.

### Correlation analysis between interaction metrics and quality score

**Fig 7:**
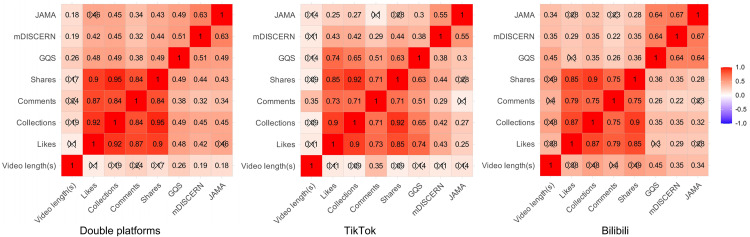
Platform Relationship Heat Map.

Fig 7 illustrates the relationship between engagement metrics and content quality scores. Analysis of the overall situation across both platforms reveals significant relationships across engagement measures, including likes, collections, comments, and shares. The three scoring measures demonstrate moderate associations with these engagement indicators (*P* < 0.05).

**Fig 8:**
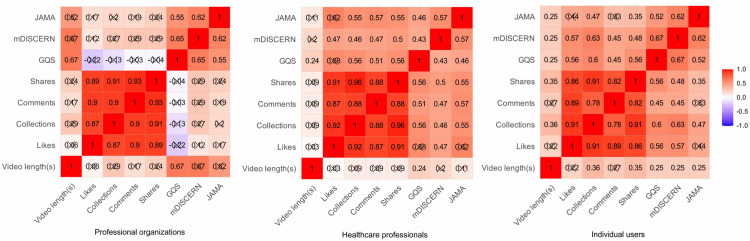
Relationship Heatmap Among Different Uploaders.

Across platforms, we find that TikTok's GQS ratings correlate more closely with engagement measures, while Bilibili's three scoring criteria have good internal consistency. User engagement patterns are highly correlated among uploaders, although professional institutions indicate a disparity between video engagement volume and quality (Fig 8).

## Discussion

This study performed a cross-sectional examination of 252 videos related to biological treatments for psoriasis, utilising social media sites as the access point. The assessment encompassed various parameters, including distribution attributes, uploader discrepancies, and material calibre. The findings demonstrated disparities in diffusion ecosystems across platforms and underscored the quality problems confronting medical information in the new media landscape.

### Differences among platforms and their communication significance

TikTok surpasses Bilibili in video volume, with a distribution of 54.76% compared to 45.24%. Interaction metrics indicate notable differences: TikTok's likes, comments, collections, and shares significantly surpass those of Bilibili, with a particularly marked disparity in likes (215.50 compared to 6.00). This demonstrates TikTok's enhanced ability for swift content distribution and extensive audience interaction. We propose that these differences likely arise from platform recommendation algorithms and user preferences [23]. TikTok viewers choose brief, fragmented content, whereas Bilibili users are more inclined towards medium-to-long-form educational films [24,25]. This is also evident in the disparity in video duration (TikTok: 78.00 seconds vs. Bilibili: 96.50 seconds). He and Ming demonstrate a clear correlation between engagement metrics and video length: concise movies have benefits including enhanced accessibility, improved comprehensibility, and increased shareability. Succinct content styles are more likely to capture users’ attention. In contrast, videos beyond 60 seconds generally garner less daily likes [26,27].

Significantly, regarding content subjects, Bilibili videos more commonly discuss medical charges (57.97% compared to 24.56%), recurrence (56.52% versus 35.09%), and medicine types (64.49% relative to 42.98%). This information enables patients to accurately assess economic constraints in relation to drug options, hence improving their capacity to manage expectations concerning treatment expenses and recurrence probabilities. Excessive focus on financial concerns or recurrence rates may induce anxiety, potentially diminishing treatment adherence and resulting in negative outcomes such as treatment cessation [28,29]. TikTok exhibits a greater percentage in elucidating disease mechanisms (36.58% compared to 17.39%), facilitating patients’ comprehension of their conditions and treatment fundamentals. This improves the scientific endorsement of standardized treatment and encourages sustained adherence over time. Insufficient information regarding costs and recurrence risks may lead to patient disappointment when faced with real-world treatment challenges, which could adversely impact their prognosis [30,31].

Both platforms exhibit unique strengths and weaknesses in their content orientation. Effective scientific medical communication must integrate the dissemination of conceptual knowledge with an awareness of practical risks at the operational level. This approach enhances patients’ scientific understanding and treatment adherence while aiding in the establishment of realistic long-term management expectations, ultimately improving treatment outcomes and prognosis [32].

### Content quality variability: Insights from mDISCERN, GQS, and JAMA scoring

Regarding quality evaluation, both platforms achieved a median GQS score of 3. TikTok markedly surpassed Bilibili in mDISCERN and JAMA scores (mDISCERN: TikTok Q1 = 3.00, Bilibili Q1 = 2.00, *P* < 0.01; JAMA median 2.00 compared to 1.00, *P* < 0.001). This suggests that, despite TikTok's emphasis on short videos, it surpasses Bilibili in overall scientific rigor and reliability [33]. This suggests that TikTok's greater percentage of healthcare professionals among its content creators (67% compared to 56%) plays a role, as those with more robust professional backgrounds are more capable of aligning their content with medical evidence and academic standards. This, consequently, improves the reliability and reference value of videos to some degree [34]. The composition of platform uploaders directly affects the distribution of information quality, while audience engagement does not necessarily have a positive correlation with quality [35,36]. The overall quality of these videos is relatively low, with most scoring moderately on the GQS scale (2–4 points). The quality of content on both platforms inadequately addresses patients’ needs for scientific information [37].

### The dual impact of uploader identity on dissemination and quality

Subsequent stratified analysis indicated that movies produced by professional organizations had the longest duration (247 seconds) and the highest quality scores (median GQS of 4.00; median JAMA of 3.00). Their superior scientific rigor and comprehensiveness can be attributed to a more professional team composition, refined video content, and robust review mechanisms [38]. Nonetheless, their involvement was the least, with a median of merely 2.00 comments—significantly lower than that of individual users (73.00). In contrast, videos published by individual users typically focused on personal experiences, medical treatment procedures, or medication experiences, with information more directly related to patient issues. These videos demonstrated the greatest interaction yet received the lowest JAMA scores (2.00 compared to 3.00 for professional organizations, *P* < 0.001). This suggests that the public more quickly connects with accessible, quotidian statements, albeit such content lacks scientific rigour [39]. Videos produced by healthcare professionals achieve a balance between expertise and effective dissemination. Their median GQS score of 3.00 and enhanced engagement metrics relative to professional organizations indicate they are a significant influence on future medical communication via social media [20]. They can provide essential scientific precision while facilitating effective communication through suitable simplification.

The stark contrast between professional organizations and individual users exposes a fundamental contradiction in medical communication: superior quality does not inherently equate to effective distribution [40,41]. We assert that professional organizations must uphold academic rigour while embracing communication styles preferred by individual users, including the use of patient case studies, testimonials, and colloquial language, to improve audience engagement.

### Correlation between interaction metrics and quality scores

Spearman correlation study demonstrated robust relationships among engagement indicators, suggesting that video popularity frequently increases concurrently [42]. Notably, Quality scores also exhibit a moderately strong positive correlation with multiple engagement metrics. This indicates that certain viewers convey their preference for high-quality information via their interactions [43]. Nonetheless, this trend is not consistently observed across different platforms: On TikTok, GQS scores show a stronger correlation with likes, collections, and shares; on Bilibili, the three quality measures exhibit significant intercorrelations, indicating that its audience may prioritise the comprehensiveness of content structure. These disparities underscore unique biases in audience perception of content and offer insights for enhancing platform recommendation systems. For TikTok, the recommendation weight for highly engaging videos should be optimized, as they may indicate superior quality and content. Bilibili can enhance its quality certification and labeling systems by implementing tags like “Professional Certification” or “Structured Premium Content,” facilitating users’ ability to swiftly identify high-quality videos [44,45]. At the same time, we must maintain vigilance against videos that may attract considerable attention due to exaggerated or sensationalised content, yet lack scientific validity [46,47]. This phenomenon was also observed during our preliminary video exclusion process. The lack of a reliable verification process on the Bilibili platform resulted in the unintended promotion of numerous videos irrelevant to this study (36 videos eliminated from the Bilibili group, 12 from the TikTok group).

Given that professional organizations produce the highest-quality videos (mDISCERN: 4.00, GQS: 4.00, and JAMA: 3.00), and considering the 0.65 correlation between GQS and video duration, we strongly recommend that patients watch longer videos released by professional organizations. Additionally, the professional organizations group demonstrated a discrepancy between interaction metrics and quality, marked by high video quality yet low engagement levels. This finding is consistent with prior research conducted by CUI and ZHANG et al [48,49]. Although they excel in scientific rigor and standardization, information released by organizations with greater professional backgrounds struggles to inspire widespread engagement and sharing due to its lack of entertainment and high barrier to understanding specialized knowledge [50]. We think such organizations should find strategies to promote scholarly dissemination while maintaining quality. Combining scientific accuracy with compelling information, improving headlines and hashtags, and publishing videos on more platforms may also help.

### Implications for clinical practice and patient education

Biological agents have emerged as a significant advancement in the treatment of psoriasis, demonstrating notable efficacy and broadening their indications [51,52]. Nonetheless, the issues of high cost, long-term safety, and immune drift continue to be subjects of significant discussion. This study indicates that the overall quality and reliability of short videos on TikTok and Bilibili platforms regarding biologic treatments for psoriasis are generally average. In comparison, TikTok and professional organizations demonstrate higher quality. Furthermore, relevant content primarily focuses on clinical manifestations, drug types, and recurrence patterns. Significant gaps exist in epidemiology, preventive measures, and long-term management content (e.g., preventive content accounted for only 19.57% and 14.04%). When patients primarily obtain information emphasizing “significant efficacy” through social media without fully understanding associated risks, costs, and recurrence rates, it may lead to unmet expectations and irrational decision-making [22,53]. For example, a lot of short-video platforms highlight visually dramatic effects like “clear skin lesions” and “rapid results” from biologic drugs, but they hardly ever discuss their high costs, the need for long-term maintenance medication, or the possibility of relapse after stopping. Some patients wrongly think that a temporary improvement in skin lesions indicates a “cure” in the absence of comprehensive medical information, which causes them to cease therapy on their own or constantly switch regimens. In the end, this causes the illness to deteriorate or repeat [54,55]. During the screening process, we also observed a subset of videos expressing skepticism toward biologic therapies or advocating alternative treatment approaches, such as exclusive reliance on traditional Chinese medicine or unverified combination therapies. Some of these videos emphasized adverse effects of biologics without providing balanced evidence or cited exaggerated risks, which may contribute to patient anxiety and reduced treatment adherence. Although such content constituted a minority of included videos, its presence reflects a broader phenomenon of oppositional or alternative narratives in online health communication. These videos often received variable engagement and tended to score lower on reliability metrics, underscoring the importance of evidence-based verification mechanisms on social media platforms.

Therefore, future medical science communication must focus on addressing these gaps: Platforms need to strengthen user verification and management, and improve the video delivery system; professional organizations should employ vivid and easily understandable presentation styles in their videos; and healthcare professionals should enhance video quality and completeness while supplementing content on epidemiology, prevention, and management. Additionally, this result has important reference value for health policy and decision-making. Based on these findings, policymakers should improve health communication tactics by enhancing instructional materials on managing psoriasis over the long term and using medications sensibly on public platforms. In order to guide multi-stakeholder collaboration (government, organizations, and media) and collectively create an accurate and balanced information ecosystem, efforts should be made concurrently to standardize and control medical science communication material. This strategy seeks to increase the effectiveness of healthcare resource allocation and improve the quality of patient decision-making.

### Limitations and future research directions

This study has several limitations. First, the use of standardized disease names and terminology during the search may lead to the omission of videos that employ slang or non-standard terms. This research employs a cross-sectional analysis, which limits its ability to account for the dynamic fluctuations in social media content, especially considering the swift updates associated with short video formats, where data may change over time. Moreover, the collection of short videos did not impose any restrictions on their publication dates, which may have affected the accuracy of some data. Furthermore, this study selected only TikTok and Bilibili as its platforms, excluding others such as YouTube and Facebook. The results are constrained by geographical or language limitations in the search data, limiting the generalizability of this research. The rating systems (mDISCERN, GQS, JAMA), despite their widespread application, continue to demonstrate subjective biases. Future research may incorporate more objective scoring criteria or AI processing technologies for the evaluation of extensive datasets. This study did not investigate audience characteristics, including variations in information reception across different age groups, educational levels, and health literacy. Future research should utilise questionnaires and audience interviews to thoroughly evaluate the acceptance and impact of medical science communication content.

## Conclusions

This study elucidates the quality and dissemination characteristics of content related to biologic therapy for psoriasis on major Chinese social media platforms. TikTok demonstrates greater dissemination capacity but carries the risk of oversimplification, whereas Bilibili provides more systematic content yet suffers from limited interactivity. Videos uploaded by professional organizations exhibit the highest reliability and transparency but the lowest engagement. After maintaining existing quality standards while enhancing engagement and accessibility, it is recommended that future efforts strongly encourage professional organizations to participate in short-form video science communication. Furthermore, adding subjects like prevention, recurrence, and epidemiology to scientific communication videos would make them more comprehensive. These findings contribute to the dissemination of high-quality medical information, enabling patients to select and manage biologic therapies in a more rational and scientifically informed manner.

## Supporting information

S1 Table S1 Appendix. (XLSX)

## Abbreviations

GQS: Global Quality Scale
JAMA: Journal of the American Medical Association
mDISCERN: modified DISCERN
IQR: Interquartile Range.
